# Superoxide dismutase as a protective factor for microalbuminuria in hypertensive patients

**DOI:** 10.1038/s41598-022-24804-3

**Published:** 2022-11-28

**Authors:** Xiaoqian Yu, Rui Xu, Wen Huang, Lin Lin, Fei Zheng, Xia Wu

**Affiliations:** 1 Department of Cardiology, Jinan Central Hospital, Shandong University, Jinan, 250013 China; 2 Department of Cardiology, Central Hospital Affiliated to Shandong First Medical University, Jinan, 250013 Shandong China; 3 Department of Medical Ultrasound, The First Affiliated Hospital of Shandong First Medical University, Jinan, 250014 China; 4 Department of Cardiology, Liaocheng People’s Hospital, Liaocheng, 252000 China; 5 Department of Cardiology, The First Affiliated Hospital of Shandong First Medical University, Jinan, 250014 China; 6 Department of Rehabilitation Medicine, The Second Hospital of Shandong University, Jinan, 250033 China

**Keywords:** Cardiology, Nephrology

## Abstract

Oxidative stress had been linked to hypertensive renal impairment in previous investigations. Superoxide dismutase (SOD) was a clinically available oxidative stress biomarker. The association between SOD and the microalbuminuria in hypertensive patients has not been established. From January 2017 to December 2018, data on 690 patients with essential hypertension were collected retrospectively at Shandong Provincial Qianfoshan Hospital. Patients were divided into hypertension with microalbuminuria group (HM) and hypertension without microalbuminuria group (NHM). Clinical data from patients were collected and compared between the two groups. *Spearman* correlation analysis was used to analyze the correlation between UACR and SOD. Univariate and multivariate logistic regression analyses were used to screen for the risk factors for HM. Our research included 556 patients in the NHM group and 134 patients in the HM group. *Spearman* correlation analysis showed a negative correlation between SOD and UACR (*P* < 0.001). Multivariate logistic regression analysis showed SOD was an independent protective factor in hypertensive patients with HM. In hypertensive patients, a substantial, negative correlation between SOD and early renal damage was found, suggesting that SOD may protect renal function.

## Introduction

In low- and middle-income nations, notably, hypertension is a major contributor to cardiovascular disease and premature death^1^. An estimated 1.28 billion people between the ages of 30 and 79 are affected with hypertension worldwide, with two-thirds of them residing in low- and middle-income nations. Unbelievably, 46% of hypertension patients are totally ignorant of their condition^2^. As a result, the harmful effects of hypertension on target organs such as heart, brain, and kidney become more obvious^3^.

Besides, hypertension is also the primary risk factor for chronic kidney disease (CKD). Hypertension coexists in approximately 80–85% of CKD and in hypertensive patients, about 15.8% have CKD^4^. According to global research, the number of individuals with all-stage CKD reached almost 700 million in 2017, resulting in 1.2 million deaths^5^.

However, CKD can be reversed in the early stage of renal damage. As a result, for a better prognosis, early detection and treatment are crucial.

According to related research, oxidative stress was the main cause of hypertension with microalbuminuria^6^, reactive oxygen species (ROS) could cause glomerular cell dysfunction, renal vascular endothelial injury, and the production of proteinuria^7^, by increasing renin release^8^, stimulating renal afferent nerve activity^9^, affecting renal arteriole systolic and perfusion pressure^10^, promoting proinflammatory signaling pathway [c-Jun N-terminal kinase(JNK), p38] and major transcription factors such as activating protein-1 (AP-1) and nuclear factor kappa-B (NF kB)^11^. Superoxide dismutase (SOD) is one of the most important antioxidants enzymes, which can specifically scavenge intracellular and extracellular superoxide in vivo by decomposing it into hydrogen peroxide and oxygen molecules. Therefore, SOD plays an important role in maintaining the balance of redox. Given the important role of SOD on the balance of redox and renal damage, we are tempted to propose that SOD maybe an independent protective factor for microalbuminuria. Its link with the microalbuminuria of hypertension (HM), however, has yet to be determined. This study was designed to investigate the relation between SOD and HM, hoping to provide a new direction to prevent and treat hypertension with microalbuminuria.

## Methods

### Study population

A total of 690 patients were included in the study based on their diagnosis of hypertension in Shandong Provincial Qianfoshan Hospital from January 2017 to December 2018. Inclusion criteria: All patients followed the 2018 ESC/ESH hypertension guidelines^12^. Exclusion criteria: (1) Urinary microalbumin/creatinine ratio (UACR) ≥ 300 mg/g; (2) Patients with secondary hypertension; (3) Patients with diabetes; (4) Renal parenchymal or vascular lesions; (5) Severe cardiac, hepatic and renal failure; (6) Tumor; (7) Cardiovascular and cerebrovascular lesions within 6 months. According to the UACR, 690 patients were classified into the HM group (134 patients, 30 mg/g ≤ UACR < 300 mg/g) and hypertension without microalbuminuria group (NHM) (556 patients, UACR < 30 mg/g). The study was approved by the ethics committee of our hospital, and all patients had given informed consent.

### General clinical data and laboratory findings

General clinical data included gender, age, smoking history, drinking history, systolic blood pressure (SBP), diastolic blood pressure (DBP), heart rate (HR), grade of hypertension, coronary atherosclerotic heart disease (CAD) and laboratory findings included ɑ_1_-microglobin (ɑ_1_-MG), urinary microalbumin (mAlb), homocysteine (Hcy), alanine aminotransferase (ALT), aspartate aminotransferase (AST), blood urea (BUN), blood uric acid (UA), serum creatinine (Scr), triglycerides (TG), retinol-binding protein (RBP), estimation of the glomerular filtration rate (eGFR); high-density lipoprotein cholesterol (HDL-c), lipoprotein-cholesterol (LDL-c), total cholesterol (TC), SOD, lipoprotein A (Lpa), blood glucose (GLU) and medication information were collected.

#### Blood pressure measurement

Clinic blood pressure was measured three times consecutively at each of the clinic visits using the HEM 7011 BP monitor (Omron Healthcare, Kyoto, Japan) after the participants had been seated for at least 5 min. Baseline clinic BP was the average of the six BP readings at the two screening visits.

#### Laboratory examination

After 12 h. of fasting, blood samples were collected from the anterior cubital vein between 8 a.m. and 10 a.m. Early-morning primary urine was considered to be morning urine. All blood and urine samples were analyzed in the laboratory of the Shandong Provincial Qianfoshan Hospital, their quality was strictly controlled. Detection of TC, LDL-C, TG, HDL-C used the blood lipid determination kit of Beijing Leadman Biochemical Technology Co., LTD, using enzyme-linked immunosorbent test (ELISA) method; SCr and BUN used the renal function biochemical kit of Shanghai Tongjing Life Technology Co., Ltd, to determine BUN by urease-glutamate dehydrogenase method and SCr by oxidase method. Detection of GLU was determined using the hexose kinase method using the glucose determination kit from Beijing Jiehui Bogao Biotech Co., Ltd. Hcy was detected by homocysteine automatic analysis detector, which was measured by Beijing mreda Technology Co., LTD, ALT, AST, ɑ_1_-MG were detected by ELISA kit (Beijing mreda Technology Co., LTD, Beijing, China). SOD (Shanghai Xinyu Biotechnology co., LTD, Shanghai, China, SOD ELISA kit), RBP (Ningbo ruiyuan biotechnology co. LTD, Ningbo, China, Turbidimetric immunoassay method; Reebio reagent). Lp(a) (Yaji Biosystems, Shanghai, China, (Lpa ELISA kit,), mAlb (Beijing Zhongshan Golden Bridge Biotechnology Co., Ltd., Beijing, China, nephelometric immunoassay). UA (Beijing Dogesce Biotechnology Co., Ltd, Beijing, China, ELISA) were detected. Estimated glomerular filtration rate was calculated by the Chronic Kidney Disease Epidemiology Collaboration (CKD-EPI) formula^13^. Urine albumin (Audit Diagnostics, Beijing mreda Technology Co., LTD, Beijing, China, immunoturbidimetric tests) and urine creatinine (Audit Diagnostics, Nanjing Caobenyuan Biotechnology Co., Ltd, Nanjing, China, immunoturbidimetric tests) were measured, and the ratio of albumin to creatinine was calculated. All detection steps and operations were carried out in accordance with the kit instructions.

### Statistical analysis

Statistical analysis was performed using the Statistical Package for the Social Science (SPSS) version 25.0 (Armonk, NY: IBM Corp). Continuous data with normal distribution were expressed as *X* ± *S*, and an independent sample t-test was used for comparisons between groups. When the data did not conform to a normal distribution, *M* (*P*_25_, *P*_75_) was used to express our data and the Mann–Whitney U test was used for comparisons. Categorical data were presented as examples and percentages and examined with ^2^ test. The association between UACR and SOD was investigated using *Spearman* correlation analysis*.* The risk factors of HM were determined by using univariate and multivariate logistic regression. *P* < 0.05 was considered as a statistically significant difference.

### Ethical Approval

The studies involving human participants were reviewed and approved by Ethics Committee of Shandong Provincial Qianfoshan Hospital. All procedures performed in studies involving human participants were in accordance with the ethical standards of the institutional and/or national research committee and with the 1964 Helsinki Declaration and its later amendments or comparable ethical standards.

## Results

Table 1 showed the study's fundamental characteristics. A total of 556 patients were in the NHM group and 134 patients were in the HM group. The average age of the study participants was 60.24 ± 11.74 years, and the gender distribution was approximately equal (49.1% males and 50.9% females). The mean values of SBP, DBP, and HR were 145.13 mmHg, 85.97 mmHg, and 73.65 beats/min, respectively.

**Table 1:** Comparison of patients’ general data.

Characteristic	NHM(N = 556)	HM(N = 134)	*P* value
Age–yr	59 ± 11	63 ± 11	0.54
Male sex-no. (%)	294 (53) *	57 (43)	0.02
SBP–mmHg	144 ± 19**	151 ± 24	< 0.01
DBP–mmHg	86 ± 14	87 ± 15	0.3
HR–beats/min	73 ± 13	75 ± 16	0.2
**Grade of hypertension–no. (%)**			
Grade1 (mild)	70 (13)	7 (5)	< 0.01
Grade2(moderate)	152 (27)	22 (16)	
Grade3(sever)	334 (60)	105 (78)	
CAD–no. (%)	380 (68)	90 (67)	0.16
Smoking history–no. (%)	169 (30)	42 (31)	0.72
Drinking history–no. (%)	205 (37) **	32 (23)	< 0.01
**Medication information–no. (%)**			
ACEI	92 (17)	26 (19)	0.13
ARB	176 (32)	42 (31)	0.93
β-blockers	277 (50)	68 (51)	0.81
CCB	229 (41)	63 (47)	0.07
Diuretics	103 (19)	23 (17)	0.12

The data are presented as mean ± SD for continuous variables and n for categorical variables.

*SBP *Systolic blood pressure, *DBP* Diastolic blood pressure, *HR* Heart rate, *CAD* Coronary atherosclerotic heart disease, *ACEI* Angiotensin converting enzyme inhibitor, *ARB* Angiotensin receptor blocker, *CCB* Calcium channel blocker.

*significantly different from the HM group (*P *< 0.05)

**significantly different from the HM group (*P *< 0.01).

### Comparison of general data and laboratory findings of patients in different groups

In the HM group, male sex, SBP, grade of hypertension, ɑ_1_-MG, mAlb, HCY, BUN, UA, Scr, RBP and GLU were significantly higher than NHM group, while SOD, drinking history and eGFR were significantly lower than NHM group (Tables 1 and 2).

**Table 2:** Comparison of patients’ laboratory findings.

Group	NHM(n = 556)	HM(n = 134)	*P* value
α1-MG [mg/L, M (*P*_*25*_, *P*_*75*_)]	5.30 (3.32, 8.64) **	11.10 (5.69, 20.44)	< 0.01
mAlb [mg/L, M (*P*_*25*_, *P*_*75*_)]	11.28 (7.81, 16.10) **	35.50 (21.47, 73.24)	< 0.01
HCY (μmol/L)	14.72 ± 7.73 *	16.81 ± 9.42	0.02
ALT [U/L, M (*P*_*25*_, *P*_*75*_)]	17.30 (12.7, 25.3)	16.80 (11.45, 26.5)	0.39
AST [U/L, M (*P*_*25*_, *P*_*75*_)]	19.30 (16.3, 24.2)	19.50 (15.8, 28.45)	0.36
BUN (mg/dL)	31.02 ± 10.2**	34.32 ± 14.34	< 0.01
UA (mg/dL)	5.38 ± 1.37**	5.53 ± 1.77	< 0.01
Scr [mg/dL, M (*P*_*25*_, *P*_*75*_)]	0.76 (0.64, 0.88) **	0.82 (0.64, 1.02)	< 0.01
TG (mg/dL)	131.12 ± 89.76	138.16 ± 88.00	0.36
RBP (mg/dL)	5.04 ± 1.25**	5.35 ± 1.65	< 0.01
LDL-c (mg/dL)	101.68 ± 31.70	92.26 ± 30.93	0.48
HDL-c (mg/dL)	44.46 ± 10.44	43.30 ± 10.05	0.50
TC (mg/dL)	170.92 ± 40.99	165.89 ± 42.54	0.56
eGFR (ml/min)	108.55 ± 24.71**	98.08 ± 38.92	< 0.01
SOD (U/mL)	168.31 ± 18.16**	163.53 ± 22.41	< 0.01
Lpa [mg/dL, M (*P*_*25*_, *P*_*75*_)]	13.03 (6.70, 25.81)	13.91 (6.95, 25.14)	0.83
GLU (mg/dL)	94.42 ± 14.76 *	97.2 ± 17.64	0.04

The data are presented as mean ± SD for continuous variables and *M* (*P*_25_, *P*_75_) for incontinuous variables, n for categorical variables.

*α1-MG* ɑ_1_-microglobin, *mAlb* Urinary microalbumin, *Hcy* Homocysteine, *ALT* Alanine aminotransferase, *AST* Aspartate aminotransferase, *BUN* Blood urea, *UA* Blood uric acid, *Scr* Serum creatinine, *TG* Triglycerides, *RBP* Retinol-binding protein, *LDL-c* Low density lipoprotein-cholesterol, *HDL-c* High-density lipoprotein cholesterol, *TC* Total cholesterol, *Egfr* Estimation of the glomerular filtration rate, *SOD*  Superoxide dismutase, *Lpa* lipoprotein A, *GLU* Blood glucose.

*significantly different from the HM group (*P *< 0.05)

**significantly different from the HM group (*P *< 0.01).

### Univariate logistic regression analysis

**Figure 1:**
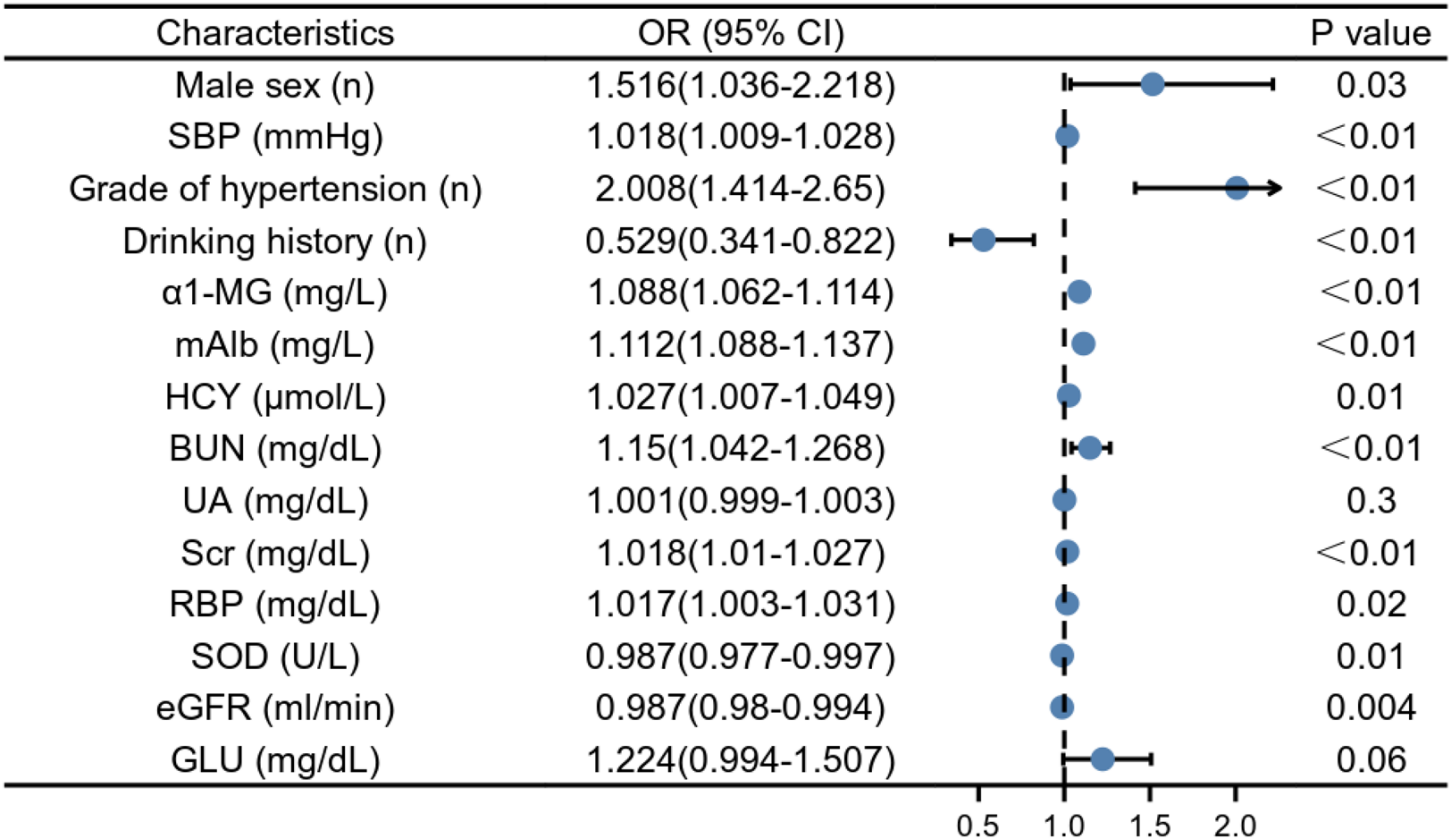
Univariate logistic analysis. *SBP* Systolic blood pressure, *α1-MG* ɑ_1_-microglobin, *mAlb* Urinary microalbumin, *HCY* Homocysteine, *BUN* Blood urea, *UA* Blood uric acid, Scr Serum creatinine, RBP Retinol-binding protein, *SOD* Superoxide dismutase, Egfr Estimation of the glomerular filtration rate, *GLU* Blood glucose.

The factors including male sex, SBP, grade of hypertension, ɑ_1_-MG, mAlb, HCY, BUN, UA, Scr, RBP, GLU, SOD, drinking history and eGFR that were statistically significant (*P* < 0.05) in the t-test were used as the independent variable, whereas the early renal damage occurred was taken as the dependent variable, establishing a univariate logistic regression model, which showed that the grade of hypertension, SBP, Hcy, Scr, RBP, mAlb, UA, Lpa were risk factors for HM, SOD, drinking history and eGFR were protective factors (Fig. 1).

### Multivariate logistic regression analysis

**Figure 2:**
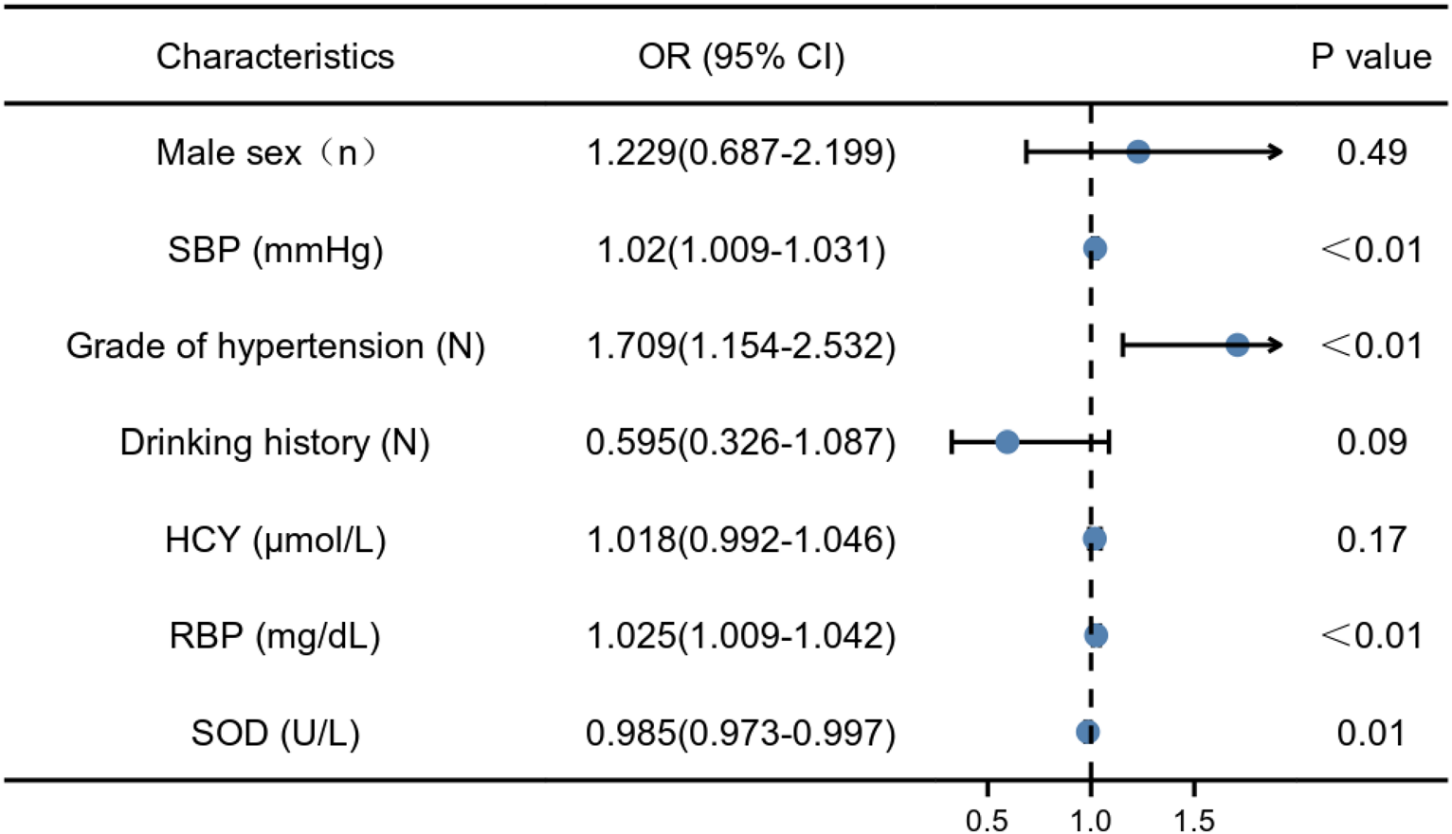
Multivariate logistic-analysis. *SBP* Systolic blood pressure, *HCY* Homocysteine, *RBP* Retinol-binding protein, *SOD* Superoxide dismutase.

Factors with *P* < 0.05 in the univariate analysis were entered into the multivariate analysis. It showed that the grade of hypertension, SBP and RBP were independent risk factors for HM, but SOD was an independent protective factor for it (Fig. 2).

### *Spearman* correlation analysis

**Figure 3:**
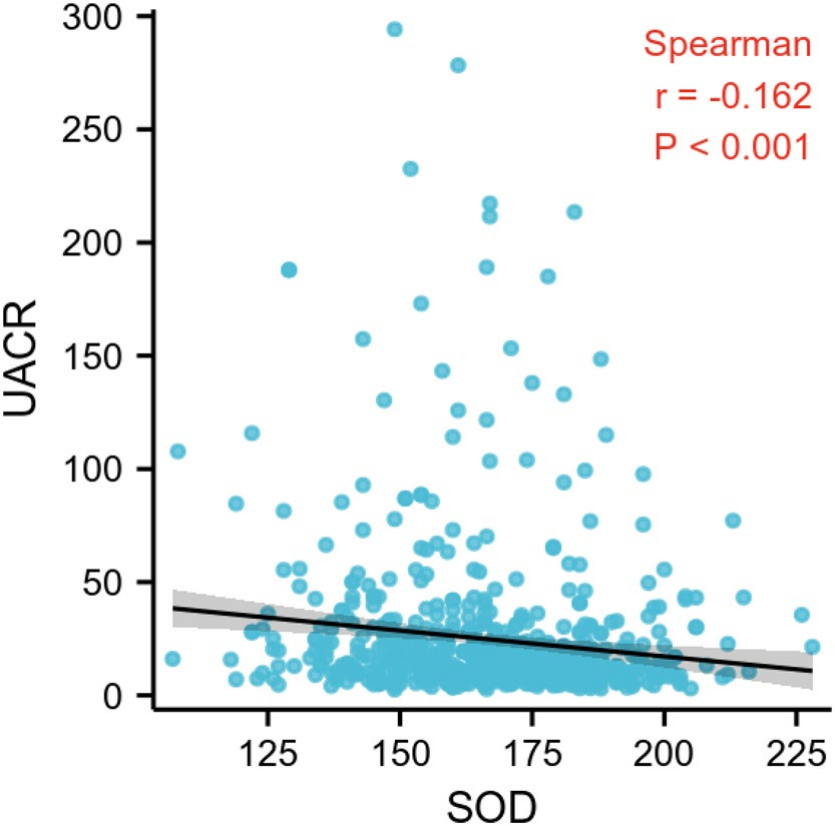
*Spearman* correlation analysis. The result of *Spearman* correlation analysis.

To further examine whether there is a linear relationship between SOD and UACR. *Spearman* correlation analysis was used in the further analysis. A negative linear relationship was found between SOD and UACR in the present study (Fig. 3).

## Discussion

Our results showed that a significant, inverse relationship between SOD and early renal damage in hypertensive patients was demonstrated, implying a possible protective role of SOD to renal function, which might provide evidence for future treatment-related studies.

The kidney is one of the most important target organs of hypertension. Mostly, the renal damage has insidiously early-onset and gradually increases as the disease progressed, which can form hypertensive nephropathy, causing an extremely poor prognosis^14^. Related studies suggested that oxidative stress was the main cause of HM, ROS can react with endothelium-derived nitric oxide (NO) to promote vasoconstriction^15^ and affect the reabsorption of sodium ions, resulting in the imbalance of water and sodium^16^. SOD is a key antioxidant enzyme in the human body that eliminates superoxide anions' cellular damaging effects^17^. When compared with the NHM group, the SOD level in the HM group was significantly lower, which was inversely proportional to UACR. Furthermore, we also found that a high level of SOD was an independent protective factor for HM, even after adjustment for other relevant predictors including mAlb, α1-MG and Scr, suggesting that SOD might be a potential therapeutic target for HM in patients with hypertension. Adler^18^ reported that reduced SOD expression led to increased oxidative stress and the development of CKD in rat animal models of spontaneous hypertension. Furthermore, inhibition of ROS with SOD mimics drug, or genetic deletion of a component of the signaling cascade, usually attenuated or delayed the onset of hypertension and preserved the kidney structure and the function^19^. By knocking down nuclear respiratory factor-1 (Nrf1), the superoxide production was further reduced, which relieved hypertension and reduced the target organ damage^20^. The rats without the SOD gene knockout produced considerably less goal-ole ROS than the SOD gene knockout rats as the pressure of renal arteriole perfusion increased^21^. Besides, SOD gene knockout rats exhibit increased vascular contractility and lowered vascular compliance in the long term, with a higher probability of vascular remodeling. On the other hand, with the decrease of superoxide, the NO reuse rate decreased, which then prevented the formation of cytotoxic proximities^22^. Interestingly, long-term administration of SOD mimetic drug could attenuate the renal effects of oxidative stress, atrophy, and fibrosis^23^, suggesting that SOD may be an important antioxidant enzyme that helps to preserve the kidneys by reducing the generation of reactive oxygen species.

At the same time, SBP and RBP were found to be independent risk factors for hypertension with HM in this study. Elevated SBP can cause renal damage by increasing the oxidative stress response and affecting the myogenic contractile effects in patients with hypertension^24^. Young^25^ found that SBP was the most common cause of impaired renal function in older individuals in a large-scale cross-sectional investigation. In a study of 998 older individuals with essential hypertension, Leng^26^ found that 24-h mean SBP was significantly associated with UACR levels, which was also in accordance with our findings. Plasma RBP is an adipokine, and the main physiological role is to transport retinol to peripheral tissues, mainly excreted through the kidney^27^. Marczak^28^ conducted a study of RBP4 and renal function levels, they found that eGFR was the most important factor affecting RBP levels. When renal damage occurred, estimation of the eGFR was reduced, which in turn led to the decrease of RBP volume in renal clearance plasma and the increase of RBP levels in plasma. Interestingly, we found that drinking was a protective factor for microalbuminuria in hypertensive patients. Although the type of drinking was not assessed, this finding may be due to the antioxidant components of alcohol drinks such as wine and beer. Future studies need to clarify the exact role of drinking in the incidence of microalbuminuria.

Our research does have certain limitations: 1. Because this is a retrospective observational study with no follow-up data, it is impossible to investigate the effect of SOD on patient outcome; 2. Due to the relatively small sample size of this study, further subgroup analysis of different blood pressure grades and liver functions is not possible; 3. This is a single-center study, and the results should be confirmed by multi-center and large samples.

To summarize, in hypertensive patients, a substantial, negative correlation between SOD and early renal damage was found, suggesting that SOD may protect renal function. SOD is a common and inexpensive index in clinic and is an important marker of oxidative stress. By analyzing its correlation with early renal damage in hypertension, it might explain the possible mechanism of hypertensive renal damage and provide a new direction for diagnosis and treatment.

## Data Availability

The data presented in this study are available on request from the corresponding author.
